# Learning temporal relationships between symbols with Laplace Neural Manifolds

**Published:** 2024-09-22

**Authors:** Marc W. Howard, Zahra Gh. Esfahani, Bao Le, Per B. Sederberg

**Affiliations:** 1 Department of Psychological and Brain Sciences, Boston University, 610 Commonwealth Ave, Boston, 02215, MA, USA.; 2 Department of Psychology, University of Virginia, 409 McCormick Road, Charlottesville, 22904, VA, USA.

**Keywords:** Temporal memory, prediction, Laplace transform, convolution

## Abstract

Firing across populations of neurons in many regions of the mammalian brain maintains a temporal memory, a neural timeline of the recent past. Behavioral results demonstrate that people can both remember the past and anticipate the future over an analogous internal timeline. This paper presents a mathematical framework for building this timeline of the future. We assume that the input to the system is a time series of symbols—sparse tokenized representations of the present—in continuous time. The goal is to record pairwise temporal relationships between symbols over a wide range of time scales. We assume that the brain has access to a temporal memory in the form of the real Laplace transform. Hebbian associations with a diversity of synaptic time scales are formed between the past timeline and the present symbol. The associative memory stores the convolution between the past and the present. Knowing the temporal relationship between the past and the present allows one to infer relationships between the present and the future. With appropriate normalization, this Hebbian associative matrix can store a Laplace successor representation and a Laplace predecessor representation from which measures of temporal contingency can be evaluated. The diversity of synaptic time constants allows for learning of non-stationary statistics as well as joint statistics between triplets of symbols. This framework synthesizes a number of recent neuroscientific findings including results from dopamine neurons in the mesolimbic forebrain.

## 1 Introduction

Consider the experience of listening to a familiar melody. As the song unfolds, notes feel as if they recede away from the present, an almost spatial experience. According to Husserl (1966) “points of temporal duration recede, as points of a stationary object in space recede when I ‘go away from the object’.” For a familiar melody, Husserl (1966) argues that events predicted in the future also have an analogous spatial extent, a phenomenon he referred to as *protention*. This experience is consistent with the hypothesis that the brain maintains an inner timeline extending from the distant past towards the present and from the present forwards into the future. In addition to introspection and phenomenological analysis, one can reach similar conclusions from examination of data in carefully controlled cognitive psychology experiments (Tiganj, Singh, Esfahani, & Howard, 2022).

The evolutionary utility of an extended timeline for future events is obvious. Knowing what will happen when in the future allows for selection of an appropriate action in the present. Indeed, much of computational neuroscience presumes that the fundamental goal of the cortex is to predict the future (Clark, 2013; Friston, 2010; Friston & Kiebel, 2009; Palmer, Marre, Berry, & Bialek, 2015; Rao & Ballard, 1999).

In AI, a great deal of research focuses on reinforcement learning (RL) algorithms that attempt to optimize future outcomes within a particular planning horizon (Dabney et al., 2020; Ke et al., 2018) without a temporal memory. From the perspective of psychology, RL is a natural extension of the Rescorla-Wagner model (Rescorla & Wagner, 1972) an associative model for classical conditioning (Schultz, Dayan, & Montague, 1997; Sutton & Barto, 1981; Waelti, Dickinson, & Schultz, 2001). Associative models describe connections between a pair of stimuli (or stimulus and an outcome etc) as a simple scalar value. In simple associative models, variables that affect the strength of an association, such as the number of pairings between stimuli, or attention, etc, must all combine to affect a single scalar value. Thus, although the strength of an association can fall off with the time between stimuli, the association itself does not actually convey information about time *per se* (C. Gallistel, 2021a).

Cognitive psychologists have argued that classical conditioning does not reflect atomic associations between stimuli, but rather explicit storage and retrieval of temporal relationships (Arcediano, Escobar, & Miller, 2005; Balsam & Gallistel, 2009; Cohen & Eichenbaum, 1993; C. Gallistel, Craig, & Shahan, 2019; Namboodiri, 2021). In this view, behavioral associations in classical conditioning reflect learning of temporal *contingencies* between stimuli, such that knowing that a particular stimulus was experienced in the present changes our expectations for the time at which an outcome will be experienced (Floeder, Jeong, Mohebi, & Namboodiri, 2024; Jeong et al., 2022). Such a theory clearly requires a temporal memory in order to learn temporal relationships between stimuli.

This paper presents a formal hypothesis for how populations of neurons could learn and express temporal relationships between symbols, ignoring similarity structure within stimuli. We assume the existence of a temporal memory expressed in the firing of neurons with an effectively continuous spectrum of time constants, forming the Laplace transform of the recent past (Atanas et al., 2023; Bright et al., 2020; Kanter, Lykken, Moser, & Moser, 2024; Tsao et al., 2018; Zuo et al., 2023). Neurophysiological results suggest that the temporal memory expressed in neural firing extends at least several minutes (Tsao et al., 2018). We additionally hypothesize a neural timeline of the future expressed as Laplace transform (Cao, Bright, & Howard, in press). The present is part of both the past and the future, so that the current symbol is simultaneously the most recent part of the past and the most imminent part of the future. Hebbian associations between the Laplace transform for the past and the present symbol store temporal relationships between symbols. In addition, a continuous spectrum of *synaptic* time scales enable learning of temporal relationships over time scales much longer than a few minutes. This spectrum of synaptic time constants also enables learning of higher-order relationships among symbols expressed as their joint statistics.

## 2 Constructing neural timelines of the past and future

We take as input a finite set of discrete symbols, x, y, etc., that are occasionally presented for an instant in continuous time. There are consistent temporal relationships between some of the symbols, such that knowing one symbol was presented at time t may provide information about the occurrence of another symbol at time t+τ. For convenience we assume that the time between repetitions of any given symbol is much longer than the temporal relationships that are to be discovered and much longer than the longest time constant $1/s_{\min}$. Much like the assumptions necessary to write out the Rescorla-Wagner model (C. Gallistel, 2021b; Namboodiri, 2021), this set of assumptions allows us to imagine that experience is segmented into a series of discrete trials and that each symbol can be presented at most once per trial. This assumption allows easy interpretation of quantities that we will derive.

### 2.1 The present

Let us take as input a vector valued function of time f(t). The notation v refers to a vector with each element a real number, $v^{\prime }$ is a transposed vector, so that $u^{\prime }v$ is the inner product, a scalar, and uv′ is the outer product, a matrix. We assume a tokenized representation between symbols, so that $y^{\prime }x=\delta_{y,x}$ where $\delta_{ij}$ is the Kronecker delta function. We write ${\text{f}}_{t}$ for the symbol available at time t. At instants t when no stimulus is presented, ${\text{f}}_{t}=0$, the zero vector. If we present a specific symbol at a specific time $t_{o}$, this adds to f(t) the basis vector for that symbol multiplied by a delta function over time centered at $t_{o}$. At most times, the input is zero. We will occasionally refer to the moment on a particular trial when x is presented, ${\text{f}}_{t}=x$ as $t_{x}$.

**Fig. 1:**
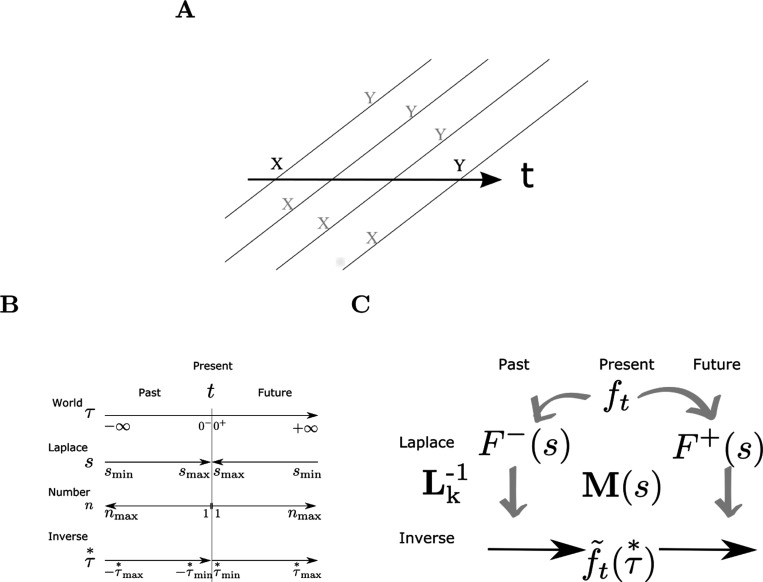
Guide to notation. **A.** Time measured externally is drawn as a horizontal line; the “internal timeline” available to the agent at each moment is drawn as a diagonal line. The remembered past at time t is drawn below the horizontal; the predicted future is drawn above the horizontal. Locations on the internal timeline are spaced to suggest logarithmic compression. Consider a case in which x and y are presented many times with a consistent temporal relationship. If x is presented at $t_{x}$ and then y is presented at some later time $t_{y}$. After x is presented at $t=t_{x}$, it recedes into the past, so for $t>t_{x}$ we find that ${\text{f}}_{t}\left(t-t_{x}\right)=x$. At the moment y is presented, $f_{t_{y}}\left(t_{y}-t_{x}\right)=x$. After the relationship between x and y is learned, then after x is presented y is predicted a time $t_{y}-t_{x}$ in the future. As time proceeds after presentation of x, the predicted occurrence of y should approach closer and closer to the present. **B.** Sign conventions. At the present moment t, objective time τ runs from −∞ to ∞. τ=0 corresponds to time t. The real Laplace domain variable s runs from 0^+^ to +∞ for both past and future, approximated as $s_{\min}$ and $s_{\max}$. The units of s are $t^{-1}$; the values corresponding to different points of the timeline are shown in the same vertical alignment. Cell number for Laplace and inverse spaces n are aligned with one another. The variable $\overset{*}{\tau }$ describes position along the inverse spaces. It is in register with τ and derived from s. **C.** The stimulus available in the present, ${\text{f}}_{t}$ provides input to two sets of neural manifolds. One set of neural manifolds represents the past; the other estimates the future. M(s) stores temporal relationships between events.

We write ${\text{f}}_{t}(\tau )$ to describe the true past that led up to time t. The continuous variable τ runs from $0^{-}$, corresponding to the moment of the past closest to the present backwards to −∞, corresponding to the distant past. Whereas ${\text{f}}_{t}$ is the symbol available in the present at a particular instant t, ${\text{f}}_{t}(\tau )$, τ∈(−∞,0) is the timeline that led up to time t. Under the assumption that every symbol is presented at most once per trial, each component of ${\text{f}}_{t}(\tau )$ over the interval τ<0 is either a delta function at some particular τ or zero everywhere. The goal of the associative memory is to provide a guess about the future that will follow time t, ${\text{f}}_{t}(\tau )$, τ∈(0,∞) (Figure 1) given the symbol available in the present.

The symbol provided in the present ${\text{f}}_{t}$ is available to both the past and the future. The present enters the past timeline at its most recent point. In this formulation, the present is also available as the most rearward portion of the future timeline. By associating the past to the rearward portion of the future, we can learn temporal relationships between symbols separated in time. By probing these associations with the present—as the most recent part of the past timeline—we can construct an extended estimate of the future.

### 2.2 Laplace neural manifolds for the past and the future

We estimate both the past and the future as functions over neural manifolds. Each manifold is a population of processing elements—neurons—each of which is indexed by a position in a coordinate space. We treat the coordinates as effectively continuous and locally Euclidean. At each moment, each neuron is mapped onto a scalar value corresponding to its firing rate over a macroscopic period of time on the order of say 100 ms. We propose that the past and the future are represented by separate manifolds that interact with one another.

The representations for both the past and the future each utilize two connected manifolds. We refer to one kind of manifold, indexed by an effectively continuous variable s, as a Laplace space. The other kind of manifold, indexed by an effectively continuous variable $\overset{*}{\tau }$, is referred to as an inverse space. Taken together, we refer to these paired representations as a Laplace Neural Manifold. The representations of the past follow previous work in theoretical neuroscience (Howard et al., 2014; Shankar & Howard, 2013), cognitive psychology (Howard, Shankar, Aue, & Criss, 2015; Salet, Kruijne, van Rijn, Los, & Meeter, 2022), and neuroscience (Bright et al., 2020; Cao, Bladon, Charczynski, Hasselmo, & Howard, 2022).

#### 2.2.1 Laplace spaces for remembered past and predicted future

The Laplace space corresponding to the past, which we write as ${\text{F}}_{t}^{-}(s)$ encodes the Laplace transform of ${\text{f}}_{t}(-\tau )$, the past leading from the present at time t back towards the infinite past:

(1) $${\text{F}}_{t}^{-}(s)=\int_{0}^{\infty }e^{-s\tau }{\text{f}}_{t}(-\tau )d\tau =\text{𝓛}\left\{{\text{f}}_{t}(-\tau )\right\}(s),\;\tau \leq 0$$

We restrict s to real values on the positive line (but see Aghajan, Kreiman, & Fried, 2023).^1^ Many neurons tile the s axis continuously for each symbol. To the extent that we can ensure a set of exponential receptive fields with a continuous spectrum of s values, we have established that ${\text{F}}_{t}^{-}(s)$ is the Laplace transform of the past. Exponential receptive fields over the past with a continuous spectrum of time constants have been observed in many brain regions and species (Atanas et al., 2023; Bright et al., 2020; Cao et al., in press; Danskin et al., 2023; Tsao et al., 2018; Zuo et al., 2023).

The index s assigned to a neuron corresponds to the inverse of its functional time constant. The Laplace space corresponding to the future, which we write as ${\text{F}}^{+}(s)$ is an attempt to estimate the Laplace transform of the future, $\text{𝓛}\left\{{\text{f}}_{t}(\tau )\right\}(s)$ over the interval τ≥0. Thus, there is a natural mapping between 1/s and |τ| within both the past and the future. By convention, s is positive for both the past and the future so that ${\text{F}}_{t}^{-}(s)$ is the Laplace transform of ${\text{f}}_{t}(-\tau )$ for τ≤0 whereas ${\text{F}}_{t}^{+}(s)$ is the Laplace transform of ${\text{f}}_{t}(\tau )$ for τ≥0.

Although s is effectively continuous, this does not require that neurons sample s evenly. Following previous work in psychology (e.g., Chater & Brown, 2008; Howard & Shankar, 2018; Piantadosi, 2016), neuroscience (Cao et al., 2022; Guo, Huson, Macosko, & Regehr, 2021), and theoretical neuroscience (Lindeberg & Fagerström, 1996; Shankar & Howard, 2013), we assume that s is sampled on a logarithmic scale. Let n be the neuron number, starting from the largest value of $s_{\max}$ nearest τ=0 and extending out from the present. We obtain a logarithmic scale by choosing ds/dn=−s.

#### 2.2.2 Updating Laplace spaces in real time

Suppose that we have arranged for one particular component of ${\text{F}}_{t}^{-}(s)$ or ${\text{F}}_{t}^{+}(s)$ to hold the Laplace transform of one particular symbol, which we write as $f_{t}(\tau )$. Suppose further that $f_{t}(\tau )$ is zero in the neighborhood of τ=0. Consider how this component, which we write as $F^{-}(s)$ or $F^{+}(s)$, should update as time passes. Let us pick some minimal increment of time Δt on the order of, say, 100 ms. At time t+Δt, information in $f_{t}(\tau )$ for τ≤0 recedes further away from the present, so that

$F_{t+\text{Δ}t}^{-}(s)=\text{𝓛}\left\{f_{t}(\tau +\text{Δ}t)\right\}$. In contrast, at time t+Δt, information in the future $f_{t}(\tau )$ for τ>0 comes closer to the present, so that $F_{t+\text{Δ}t}^{+}(s)=\text{𝓛}\left\{f_{t}(\tau -\text{Δ}t)\right\}$. More generally, suppose that $F_{t}(s)$ is the Laplace transform of a function over some variablex, $F_{t}(s)=\text{𝓛}\left\{f_{t}(x)\right\}(s)$. Defining $\alpha \equiv \frac{\text{Δ}x}{\text{Δ}t}$, we can update $F_{t}(s)$ as

(2) $$F_{t+\text{Δ}t}(s)=\text{𝓛}\left\{{\text{𝓣}}_{\alpha \text{Δ}t}f_{t}(x)\right\}(s)=e^{-s\alpha \text{Δ}t}F_{t}(s)$$

where 𝓣 is the translation operator, ${\text{𝓣}}_{a}f(x)=f(x+a)$ and we have used the expression for the Laplace transform of translated functions. Equation 2 describes a recipe for updating both $F_{t}^{\pm }(s)$ with $\alpha_{\pm }$ in the absence of new input. Using the sign convention developed here, we fix $\alpha_{-}=1$ for $F^{-}(s)$ and fix $\alpha_{+}=-1$ for $F^{+}(s)$. It is possible to incorporate changes into the rate of flow of subjective time by letting $\alpha_{\pm }$ change in register, such that $\alpha_{+}(t)=-\alpha_{-}(t)$ for all t. The expression in Eq. 2 holds more generally and can be used to update Laplace transforms over many continuous variables of interest for cognitive neuroscience (Howard & Hasselmo, 2020; Howard, Luzardo, & Tiganj, 2018; Howard et al., 2014).

We are in a position to explain how ${\text{F}}_{t}^{-}(s)$ comes to represent the Laplace transform of ${\text{f}}_{t}(-\tau )$ over the interval τ∈(−∞,0); a discussion of how ${\text{F}}_{t}^{+}(s)$ comes to estimate the future requires more development and will be postponed. When a symbol is presented at time t, it enters timeline of the past at τ=0. So, incorporating the Laplace transform of the most recent part of the past with the past that is already available and then evolving to time t+Δt we have

(3) $${\text{F}}_{t+\text{Δ}t}^{-}(s)=e^{-s\text{Δ}t}\left[{\text{F}}_{t}^{-}(s)+\text{𝓛}\left\{\delta (0){\text{f}}_{t}\right\}\right]\;\;=e^{-s\text{Δ}t}{\text{F}}_{t}^{-}(s)+e^{-s\text{Δ}t}{\text{f}}_{t}.$$

At time t+Δt, the input from time t is encoded as the Laplace transform of that symbol a time Δt in the past. At each subsequent time step, an additional factor of $e^{-s\text{Δ}t}$ accumulates. As time passes, the input from time t is always stored as Laplace transform of a delta function at the appropriate place on the timeline. Because this is true for all stimuli that enter ${\text{F}}^{-}(s)$, we conclude that ${\text{F}}_{t}^{-}(s)$ encodes the Laplace transform of the past ${\text{f}}_{t}(-\tau )$ over the interval τ≤0.

**Fig. 2:**
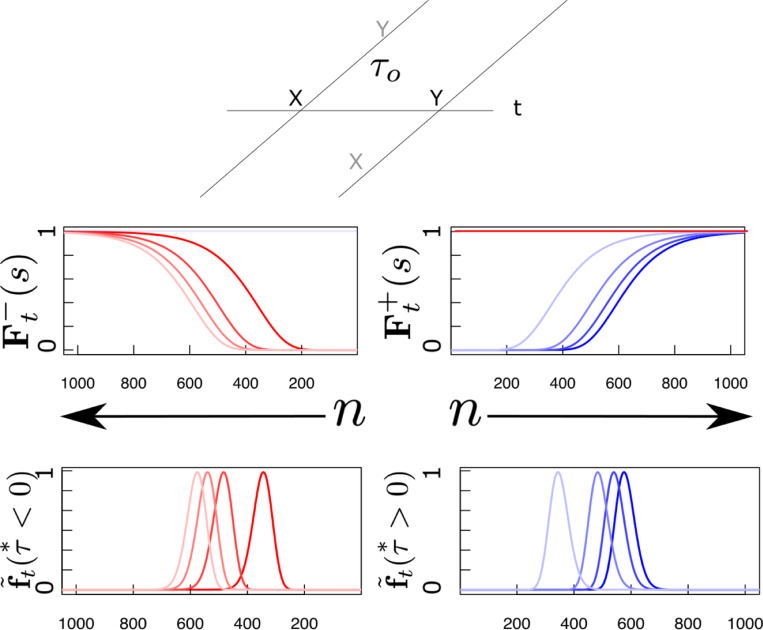
Neural manifolds that construct a logarithmically-compressed internal timeline of the past and the future. Top: A temporal relationship exists between x and y such that y always follows x after a delay of $\tau_{o}$ seconds. Consider how the internal timeline ought to behave after x is presented at t=0. At time t, the past should include x t seconds in the past and y $\tau_{o}-t$ seconds in the future. Middle and bottom: Samples of the timeline at evenly-spaced moments between zero and $\tau_{o}$. At each moment, there is a pattern of activity over neurons indexed by n. The state of the timeline at earlier moments, closer to t=0, are darker and later moments closer to $t=\tau_{o}$ are lighter. Red lines are neurons coding for x (primarily in the past except precisely at t), blue lines are neurons coding for y (primarily in the future except precisely at $t=\tau_{o}$). Middle: Laplace spaces for the past (left) and future (right) shown as a function of cell number n; Bottom: inverse spaces, constructed using the Post approximation, for the past (left) and future (right) shown as a function of log time. Exactly at time t=0, x is available a time 0^+^ in the future (dark horizontal red line, middle right). Similarly, exactly at $t=\tau_{o}$, y is available a time 0^−^ in the past (light horizontal blue line, middle left).

The middle panel of Figure 2 illustrates the profile of activity over ${\text{F}}_{t}^{-}$ and ${\text{F}}_{t}^{+}$, shown as a function of cell number n, resulting from the Laplace transform of a delta function at various moments in time. In the middle panel, the s axis for the past is reversed to allow appreciation of the relationship between past time τ≤0 and $F^{-}$. Note that the Laplace transform of a delta function has a characteristic shape as a function of cell number that merely translates as time passes. Note that the magnitude of the translation of $F^{\pm }[n]$ depends on the value of $\tau_{o}$. It can be shown that for a delta function $F_{t+\text{Δ}t}^{\pm }[n]=F_{t}^{\pm }[n+\text{Δ}n]$ with $\text{Δ}n=\alpha_{\pm }\frac{\text{Δ}t}{\tau_{o}}$. This can be appreciated by noting that the distances between successive lines in the middle panel of Fig. 2 are not constant despite the fact that they correspond to the same time displacement. Whereas Δn goes down as time passes for $F^{-}[n]$ as the past becomes more remote from the present, Δn increases with the passage of time for $F^{+}[n]$ as the future grows closer to the present.

There are implementational challenges to building a neural circuit that obeys Eq. 2; these challenges are especially serious when α<0, which requires activation to grow exponentially. If one is willing to restrict the representation of each symbol to the Laplace transform of a delta function at a single point in time, it is straightforward to implement a continuous attractor network (Khona & Fiete, 2021) to allow the “edge” in the Laplace transform as a function of n to translate appropriately. Daniels and Howard (submitted) constructed a simple continuous attractor network to demonstrate the feasibility of this approach.

#### 2.2.3 Inverse spaces for remembered past and predicted future

The mammalian brain also contains “time cells” with circumscribed receptive fields (MacDonald, Lepage, Eden, & Eichenbaum, 2011; Pastalkova, Itskov, Amarasingham, & Buzsaki, 2008; Schonhaut, Aghajan, Kahana, & Fried, 2023; Tiganj, Cromer, Roy, Miller, & Howard, 2018). Time cells resemble a “direct” estimate of the past and are reasonably well approximated as:

(4) $${\tilde{f}}_{t}(\overset{*}{\tau })=\int_{0}^{\infty }\text{Φ}\left(\frac{\tau }{\overset{*}{\tau }}\right)f_{t}(-\tau )d\tau$$

where Φ(x) is a unimodal function with its maximum at 1 and $\overset{*}{\tau }$ is here defined to be negative (Fig. 1). ${\tilde{f}}_{t}\left(\overset{*}{\tau }\right)$ estimates the true past in the neighborhood of $\overset{*}{\tau }$. As Φ() becomes more and more sharp, approaching a delta function, ${\tilde{f}}_{t}\left(\overset{*}{\tau }\right)$ goes to $f_{t}\left(\overset{*}{\tau }\right)$. In this sense $\tilde{f}\left(\overset{*}{\tau }\right)$ is like the inverse Laplace transform of $F_{t}(s)$. However, because receptive fields depend only on the ratio of $\tau /\tau^{*}$, and because neurons sample the $\overset{*}{\tau }$ axis logarithmically, $\tilde{f}(n)$ is a convolution of f(logτ) and another function of log τ that controls the blur.

The bottom panel of Figure 2 shows a graphical depiction of the inverse space for the past and the future during the interval between presentation of x and y. The inverse spaces approximate the past, ${\tilde{f}}_{t}(\overset{*}{\tau })$ for $\overset{*}{\tau }<0$ and the future, ${\tilde{f}}_{t}\left(\overset{*}{\tau }\right)$ for $\overset{*}{\tau }>0$ on a log scale. As the delta function corresponding to the time of x recedes into the past, the corresponding bump of activity in $x^{\prime }{\tilde{f}}_{t}(n)$ also moves, keeping its shape but moving more and more slowly as x recedes further and further into the past. In the future, the delta function corresponding to the predicted time of y should start a time $\tau_{o}$ in the future and come closer to the present as time passes. As the prediction for y approaches the present, the corresponding bump of activity in $y^{\prime }{\tilde{f}}_{t}(n)$ keeps its shape but the speed of the bump accelerates rather than slowing with the passage of time.

It is in principle possible to construct the inverse space from the Laplace space *via* a linear feedforward operator. Previous papers (e.g., Shankar & Howard, 2013) have made use of the Post approximation to the inverse Laplace transform to construct the inverse space from the Laplace space. This is not neurally reasonable (Gosmann, 2018); the Post approximation is difficult to implement even in artificial neural networks (e.g., Jacques, Tiganj, Howard, & Sederberg, 2021; Tano, Dayan, & Pouget, 2020). A more robust approach would be a continuous attractor network (for a review see Khona & Fiete, 2021) that takes input as the derivative of F with respect to n. The width of the bump in $\tilde{f}$ would depend on internal connections between neurons in $\tilde{f}$ and global inhibition would stabilize the activity over $\tilde{f}$. In this case, moving the bump in different directions, corresponding to α>0 and α<0 is analogous to moving a bump of activity in a ring attractor in different directions. A companion paper fleshes out these ideas (Daniels & Howard, submitted).

### 2.3 Predicting the future from the past

The previous subsection describes how to evolve the Laplace manifold for the past. If we could somehow initialize the representation of the future appropriately then we could use the same approach to evolve the Laplace manifold for the future during periods when no symbol is experienced. Initializing the future will be accomplished *via* learned temporal relationships between the past and the future.

The model has access to the Laplace transform of the past, as described above. We define the present so that it overlaps with both the most recent part of the past and the most imminent, or “rearward,” part of the future. We form Hebbian associations between the Laplace transform of the past and the Laplace transform of the rearward portion of the future. Recall that products of Laplace transforms are the Laplace transform of the convolution of these functions. Because there is a reflection between the definition of ${\text{F}}_{t}^{-}(s)$ and ${\text{F}}_{t}^{+}(s)$, the convolution of these two functions measures distances between time points in the past and the present. Later the present stimulus, taken as the Laplace transform of the most recent part of the past, can be used to recover the Laplace transform of an extended future timeline.

There are two sets of weights storing these associations, M(s) and $\bar{M}(s)$. Each of these weights learn associations between the Laplace transform of the past, ${\text{F}}_{t}^{-}(s)$, and the present stimulus ${\text{f}}_{t}$. The two sets of weights are normalized differently. Roughly speaking, M(s) stores the Laplace transform of the future conditionalized on the present symbol. In contrast $\bar{M}(s)$ stores the Laplace transform of the past conditionalized on the present symbol. With the assumptions that let us consider discrete trials, these transforms are understandable as pairwise statistics of events corresponding to a presentation of each symbol on a trial. We will see that taken together M(s) and $\bar{M}(s)$ enable us to estimate the associative and temporal contingency between each pair of symbols conditionalized on each other symbol.

The learning rate and forgetting rate for the sets of weights fixes a time horizon for learning over trials. By choosing a continuous spectrum of forgetting rates ρ and learning rates 1−ρ, both M(ρ,s) and $\bar{M}(\rho ,s)$ retain a memory for the history as a function of trials. Continuous forgetting allows the weights to implement a discrete approximation to the Laplace transform. This property of M(ρ,s) and $\bar{M}(\rho ,s)$ means that it is in principle possible to aggregate joint statistics between stimuli.

#### 2.3.1 Encoding M(s)

The moment a nonzero stimulus ${\text{f}}_{t}$ is experienced, we assume it is available to both ${\text{F}}^{-}$ and ${\text{F}}^{+}$, triggering a number of operations which presumably occur sequentially within a small window of time on the order of 100 ms. First, the present stimulus updates a prediction for the future *via* a set of connections M organized by s. Then these connections are updated by associating the past to the present. Finally the present stimulus is added to the representation of the past. For ease of exposition we will first focus on describing the connections between the past and the future.

**Fig. 3:**
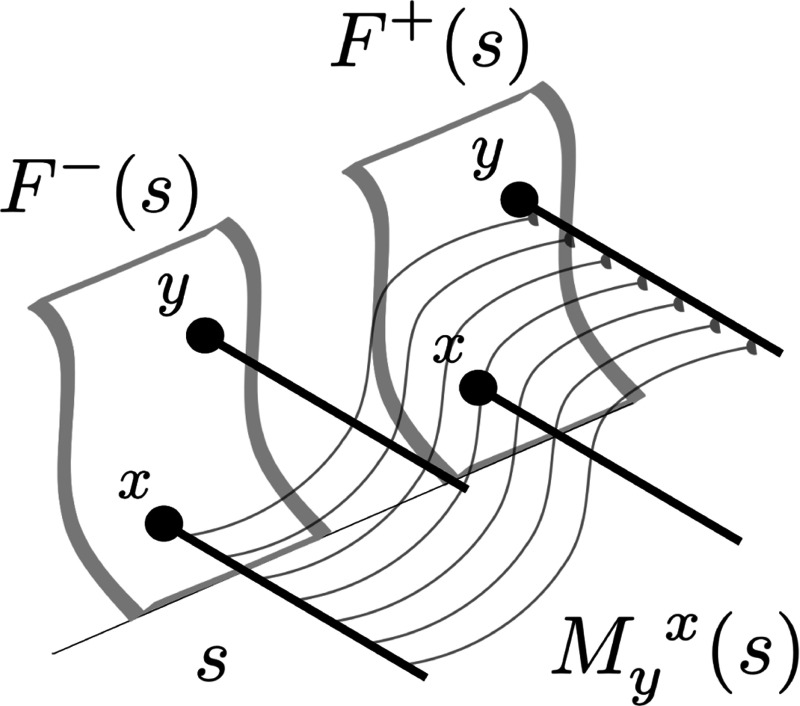
Schematic figure illustrating $M_{y}^{x}(s)$. $F^{-}(s)$ and $F^{+}(s)$ components for all the possible symbols, here shown schematically as sheets. Two symbols x and y are shown in both $F^{-}(s)$ and $F^{+}(s)$. Each symbol is associated with a population of neurons spanning a continuous set of s values, shown as the heavy lines in this cartoon. M(s) describes the connections between each symbol in ${\text{F}}^{-}(s)$ to each symbol in ${\text{F}}^{+}(s)$ for each value of s. The curved lines $M_{y}^{x}(s)$ illustrate the set of weights connecting units corresponding to x in $F^{-}$ to units corresponding y in $F^{+}$. Connections exist only between units with the same values of s. The strength of the connections in $M_{y}^{x}(s)$ vary as a function of s in a way that reflects the pairwise history between x and y.

We write M(s) for a set of connections that associates the Laplace transform of the past to the Laplace transform of the future (Fig. 3). We postpone discussion of the other set of weights $\bar{M}(s)$. For any particular value $s_{o}$, $\text{M}\left(s_{o}\right)$ is a matrix describing connections from each symbol in ${\text{F}}^{-}\left(s_{o}\right)$ to each symbol in ${\text{F}}^{+}\left(s_{o}\right)$. For each pair of symbols, say x and y, we write $M_{y}^{x}\left(s_{o}\right)$ for the strength of the connection *from* the cell corresponding to x with $s=s_{o}$ in ${\text{F}}^{-}$ *to* the cell corresponding to y in ${\text{F}}^{+}$ with $s=s_{o}$. M(s) does not include connections between neurons with different values of s. On occasion it will be useful to think of the set of connections between a pair of symbols over all values of s, which we write as $M_{y}^{x}(s)$. Similarly, we write ${\text{M}}^{y}(s)$ for the set of connections *from* y in $F^{-}$ to all stimuli in $F^{+}$ over all values of s. We write ${\text{M}}_{y}(s)$ for the set of connections *to* y in $F^{+}$ from all symbols and all values of s. In this paper, the superscripting and subscripting of $M_{y}^{x}(s)(s)$ has no significance beyond a visual aid to help keep the indices straight.

When a particular stimulus y is presented the connections to and from that stimulus in M(s) are updated. When y is presented, the connections from y in the past towards all stimuli in the present are updated as

(5) $${\text{M}}^{y}(s)\to \rho {\text{M}}^{y}(s)$$

That is, the connections from y to every other stimulus for each value of s are all scaled down by a value ρ. Later we will consider the implications of a continuous spectrum of ρ values; for now let us just treat ρ as a fixed parameter restricted to be between zero and one. When y is presented, it momentarily becomes available at the “rearward part” of the future. In much the same way that the present enters the past (Eq. 3) at $\tau =0^{-}$, we also assume that the present is also available momentarily in the future at τ=0. When y is presented, the connections from each symbol in the past to y in the future are updated as

(6) $${\text{M}}_{y}(s)\to {\text{M}}_{y}(s)+(1-\rho ){\text{F}}_{t}^{-}(s)$$

**Fig. 4:**
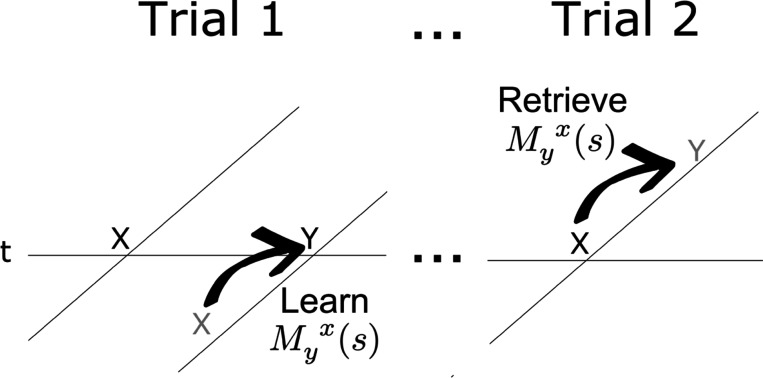
Learning and expressing pairwise associations with M(s). The horizontal line is time; the diagonal lines indicate the internal timeline at the moments they intersect. Memory for the past is below the horizontal line; prediction of the future is above. When x is presented for the first time, it predicts nothing. When y is presented, the past contains a memory for x in the past. When y is presented, $M_{y}^{x}(s)$ stores the temporal relationship between x in the past and y in the present—the rearward part of the future. In addition to storing learned relationships, connections from each item decay each time it was presented (not shown). When x is repeated much later in time, the stored connections in $M_{y}^{x}(s)$ retrieve a prediction of y in the future.

Connections involving symbols that are not present in the history retained by ${\text{F}}_{t}^{-}(s)$ are not updated. We can understand Eq. 6 as a Hebbian association between the units in ${\text{F}}^{-}(s)$, whose current activation is given by ${\text{F}}_{t}^{-}(s)$ and the units in the future ${\text{F}}^{+}(s)$ corresponding to the present stimulus y (see Fig. 4). More generally, we can understand this learning rule as strengthening connections from the past ${\text{F}}_{t}^{-}(s)$ to the rearward part of the future, $\text{𝓛}\{\delta (0)\}(s){\text{f}}_{t}=e^{-s0}{\text{f}}_{t}={\text{f}}_{t}$. Because the second term is the product of two Laplace transforms, it can also be understood as the Laplace transform of a convolution, here, the convolution of the present with the past.^2^ Convolution has long been used as an associative operation in mathematical psychology (Jones & Mewhort, 2007; Kato & Caplan, 2017; Murdock, 1982), neural networks (Blouw, Solodkin, Thagard, & Eliasmith, 2016; Eliasmith, 2013; Plate, 1995), and computational neuroscience (Steinberg & Sompolinsky, 2022).

#### 2.2.2 M(s) is a Laplace successor representation

From examination of Eqs. 5 and 6, we see that after each trial $M_{y}^{x}(s)$ is multiplied by ρ when x was presented. For trials on which y was also presented, $(1-\rho )e^{-s\tau_{o}}$ is added to $M_{y}^{x}(s)$. Writing h[i] as an indicator variable for the history of presentations of y on the trial i steps in the past we find at the conclusion of a trial that

(7) $$M_{y}^{x}(s)=(1-\rho )e^{-s\tau_{o}}\sum_{i}\rho^{i}h[i].$$

Note that if P(y∣x)=1, then after an infinitely long series of trials $\sum_{i}h[i]\rho^{i}=\frac{1}{1-\rho }$ and $M_{y}^{x}(s)=e^{-s\tau_{o}}$ for all choices of ρ. Following similar logic, if we relax the assumption that P(y∣x)=1 and take the limit as ρ goes to 1, we find that $M_{y}^{x}(s)=P(y|x)e^{-s\tau_{o}}$.

Now let us relax the assumption that the time lag between x and y always takes the same value. Let the lag be a random variable $\tau_{xy}$ subject to the constraint that $\tau_{xy}$ is always >0. This is not a fundamental restriction; if $\tau_{xy}$ changed sign, those observations would contribute to $M_{x}^{y}(s)$ instead of $M_{y}^{x}(s)$. Now, again taking the limit as ρ→1, we find

(8) $$M_{y}^{x}(s)=P(y|x)E\left[e^{-s\tau_{xy}}\right]=P(y|x)\text{𝓛}\left\{\tau_{xy}\right\}(s)$$

where we have used the definition for the Laplace transform of a random variable, again with the understanding that we restrict s to be real and positive.

Equation 8 illustrates several important properties of M(s). First, we can see that $M_{y}^{x}(s)$ provides complete information about the distribution of temporal lags between x preceding y. This can be further appreciated by noting that the Laplace transform of the random variable on the right hand side is the moment generating function of $-\tau_{xy}$. Keeping the computation in the Laplace domain means that there is no blur introduced by going into the inverse space as in previous attempts to build a model for predicting the future (Goh, Ursekar, & Howard, 2022; Shankar, Singh, & Howard, 2016; Tiganj, Gershman, Sederberg, & Howard, 2019). Second, because $\text{𝓛}\left\{\tau_{xy}\right\}(s=0)=1$ as long as the expectation of $\tau_{xy}$ is finite, $M_{y}^{x}(s=0)=P(y|x)$ and M(s=0) captures the pairwise probabilities between all symbols.

In the limit as ρ→1, M(s) is closely related to the successor representation (Carvalho, Tomov, de Cothi, Barry, & Gershman, 2024; Dayan, 1993; Gershman, Moore, Todd, Norman, & Sederberg, 2012; Momennejad et al., 2017; Stachenfeld, Botvinick, & Gershman, 2017) with a continuous distribution of discount rates (Kurth-Nelson & Redish, 2009; Masset et al., 2023; Momennejad & Howard, 2018; Sousa et al., 2023; Tano et al., 2020). More precisely, if one assumes a complete compound serial representation of the past and a fixed action policy, then computes the successor representation from RL (Dayan, 1993; Gershman et al., 2012), but with a continuous spectrum of discount rates γ, one would obtain M(s) with the identification s=−logγ. However, computing M(s) does not require temporal difference learning. In RL language, ${\text{F}}^{-}(s)$ is an ensemble of eligibility traces with a continuous spectrum of forgetting rates. Associating this multiscale eligibility trace to outcomes is sufficient to compute M(s), which we might refer to as a Laplace successor representation.

#### 2.3.3 $\bar{\text{M}}(s)$ is a Laplace predecessor representation

It is straightforward to construct a Laplace predecessor representation (Namboodiri & Stuber, 2021) using ${\text{F}}^{-}(s)$, the Laplace transform of the past, and Hebbian learning. We write out a new set of connections $\bar{M}(s)$. Adapting Eqs. 5 and 6, when each item y is presented

(9) $${\bar{M}}^{y}(s)\to \rho {\bar{M}}^{y}(s)+(1-\rho ){\text{F}}_{t}^{-}(s)$$

That is, when y is presented at time t and x is available in ${\text{F}}_{t}^{-}(s)$, ${\bar{M}}_{x}^{y}(s)$ is incremented. Following similar steps as for M(s), in the limit as ρ→1, we get

(10) $${\bar{M}}_{x}^{y}(s)=P(x|y)\text{𝓛}\left\{\tau_{xy}\right\}(s),$$

which can be compared to Equation 8. Thus, with learning as in Eq. 9, we can refer to $\bar{M}(s)$ as a Laplace predecessor representation.

Note that the convention of $\bar{M}(s)$ is different than M(s). Whereas $M_{y}^{x}(s)$ describes relationships between x preceding y, ${\bar{M}}_{y}^{x}(s)$ describes relationships between y preceding x. In this sense $\bar{M}(s)$ is like ${\text{M}}^{T}(s)$. In addition one must also account for the reflection operator involved in the definition of ${\text{F}}_{t}^{-}(s)$ as compared to ${\text{F}}_{t}^{+}(s)$ and the different marginalization.

The foregoing makes clear that if the brain has access to ${\text{F}}^{-}(s)$—an eligibility trace with a continuum of time horizons—it is straightforward to compute either a successor representation or a predecessor representation in a way that maintains complete information about the temporal relationships between stimuli. This approach does not require selecting a single time horizon or time constant for either representation (Floeder et al., 2024).

#### 2.3.4 Measures of contingency using M(s) and $\bar{M}(s)$

Information contained in M(s) and $\bar{M}(s)$ can be used to not only describe pairwise relationships between stimuli but also to assess contingency between symbols, allowing solutions to the temporal credit assignment problem. The goal here is not to propose a specific measure of contingency—there are undoubtedly a multiplicity of such rules that could be used for cognitive and neural modeling—but simply to sketch out the properties of M(s) and $\bar{M}(s)$. We continue attending to the limit as ρ→1.

For this illustration, let us restrict our attention to relationships between three symbols x, y and z. We assume for simplicity that, if they are presented on a trial, the three stimuli are presented in order on each trial. Let us refer to the time lags between symbols as random variables $\tau_{xy}$, $\tau_{yz}$; on trials where all three symbols are observed $\tau_{xz}=\tau_{xy}+\tau_{yz}$. For convenience let’s assume that the distributions are chosen such that the relative times of presentation do not overlap. We denote the probabilities of each symbol occuring on a trial such that P(z∣y) gives the conditional probability that z is observed on a trial given that y is also observed on that trial.

**Fig. 5:**
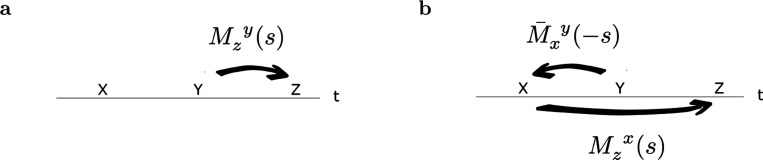
Measuring contingency by comparing pairwise relationships between y and z to pairwise relationships conditionalized on x. **a.** Equation 12 captures the Laplace transform of the random variable $\tau_{yz}$. By assumption, on each trial $\tau_{yz}=\tau_{xz}-\tau_{xy}$. **b.** Equation 14 captures the convolution of $\tau_{xz}$ and $-\tau_{xy}$. If these intervals are independent across trials then $M_{z}^{y}(s)=M_{z}^{x}(s){\bar{M}}_{x}^{y}(-s)$.

We are interested in how much “credit” to allocate y for the occurrence and timing of z, taking into account x. We will compare $M_{z}^{y}(s)$, which describes the future occurrences of z conditionalized on y in the present to $M_{z}^{x}(s){\bar{M}}_{x}^{y}(-s)$ (Fig. 5). This quantity is $M_{z}^{x}(s)$—the future of z predicted by x—multiplied by ${\bar{M}}_{x}^{y}(-s)$—the past occurrence of x predicted by knowing that y is in the present. That is, $M_{z}^{x}(s){\bar{M}}_{x}^{y}(-s)$ describes the future of z predicted by the past occurrence of x that is observed when y is in the present. The reflection operator allows the integration of these two timelines in a way that can be compared to the future of z given that y was observed in the present (Cole, Barnet, & Miller, 1995).

We will work through the implications of this high level description under very simple circumstances. Recall that under the circumstances described in this subsection,

(11) $$M_{z}^{y}(s)=P(z|y)\;\text{𝓛}\left\{\tau_{yz}\right\}(s)$$

(12) $$\;=P(z|y)\;\text{𝓛}\left\{\tau_{xz}-\tau_{xy}\right\}(s)$$

Using properties of the Laplace transform we can rewrite $M_{z}^{x}(s){\bar{M}}_{y}^{x}(-s)$ as

(13) $$M_{z}^{x}(s){\bar{M}}_{y}^{x}(-s)=P(z|x)\text{𝓛}\left\{\tau_{xz}\right\}(s)P(x|y)\text{𝓛}\left\{-\tau_{xy}\right\}(s)$$

(14) $$\;=P(z|x)P(x|y)\;\text{𝓛}\left\{\tau_{xz}*\left(-\tau_{xy}\right)\right\}(s).$$

The second term describes the Laplace transform of the convolution of $\tau_{xz}$ and $-\tau_{xy}$. Because the sum of two independent random variables is equal to their convolution, the Laplace transforms in Eqs 12 and 14 will enable us to assess the dependence between the times of presentations of x,y, and z.

##### Associative contingency at s=0

M(s=0) gives information about the pairwise probabilities between each pair of symbols. Suppose that x, y and z occur on different trials. Is the occurrence of z predicted by y or x? Or some more complex situation?

From Eqs. 8 and 10 and basic properties of random variables, we could compare

(15) $$M_{z}^{y}(s=0)=P(z|y)$$

to

(16) $$M_{z}^{x}(s=0){\bar{M}}_{x}^{y}(s=0)=P(z|x)P(x|y)$$

If Eqs 15 and 16 are equal to one another, then credit for z should go to x rather than y. To the extent they differ, then y should get credit for the occurrence of z.

Of course there are limits to how well the future can be predicted with pairwise statistics. More generally, we would like to consider joint statistics. This requires estimating higher order probabilities, e.g., P(x,z∣y). We establish later that joint statistics can be estimated from M(ρ,s). In an environment where joint statistics are important, predicting the future using simple pairwise relationships is untenable. However, it should be possible to recode the symbols into a new set of symbols that can be used to predict the future using pairwise relationships.

##### Temporal contingency

So that we can focus on temporal contingency, let us assome that all three stimuli are presented on each trial so that P(y∣x)=P(z∣y)=P(z∣x)=1. Because $M_{y}^{x}(s)$ contains information about every moment of the distribution $\tau_{xy}$ it is straightforward to ask whether the distribution of times for z conditionalized on y is higher or lower entropy than the distribution conditionalized on x. It is also possible to use M(s) and $\bar{M}(s)$ to capture more subtle temporal relationships.

Recall that the distribution of the sum of two random variables equals the convolution of those random variables if they are independent of one another. Thus comparing the distribution of $\tau_{yz}=\tau_{xz}-\tau_{xy}$ to the distribution of the convolution $\tau_{xz}*\left(-\tau_{xy}\right)$ allows us to assess the dependency across trials of the timing of the three stimuli. Equation 12 shows that the Laplace transform of $\tau_{xz}-\tau_{xy}$ is stored in $M_{z}^{y}(s)$, whereas Equation 14 shows that the Laplace transform of $\tau_{xz}-\tau_{xy}$ is stored in $M_{z}^{x}(s){\bar{M}}_{x}^{y}(-s)$. Comparing these two quantities allows us to assess the dependence between the times of occurrence of x and z conditionalized on y in the present.

### 2.4 Continuum of ρ allows a temporal memory *across* trials

For the past several subsections we have considered the limit where ρ→1. That limit is not physically realizable. How should we choose the value of ρ? The answer is that we should not choose a single value of ρ. In much the same way we treat s as a continuous variable rather than treating it as a parameter to be estimated from the data, we can also treat ρ as a continuous variable. Continuous s means that ${\text{F}}^{-}(s)$ maintains a temporal memory of the entire past. Similarly, continuous ρ enables M(ρ,s) to retain complete information about pairwise relationships as a function of trial history. Similar relationships can be worked out for $\bar{M}(\rho ,s)$ but we focus on M(ρ,s) here for simplicity.

Equation 7, which describes the situation where $\tau_{xy}$ is equal to $\tau_{o}$ on each trial, can be rewritten as

$$M_{y}^{x}(\rho ,s)=(1-\rho )e^{-s\tau_{o}}\text{𝓩}\{h[i]\}\left(\rho^{-1}\right)$$

where 𝓩{}(z) is the Z-transform, the discrete analog of the Laplace transform (Ogata, 1970). An analogous relationship can be written for $\bar{M}(s)$.

Although the notation is a bit more unwieldy, allowing $\tau_{xy}$ to vary across trials we see that the trial history of timing is also retained by M(ρ,s). Writing the delay between x and y on the trial i steps in the past as τ[i], and $H[i](s)\equiv h[i]e^{-s\tau [i]}$ we can write

(17) $$M_{y}^{x}(\rho ,s)=(1-\rho )\text{𝓩}\{H[i](s)\}\left(\rho^{-1}\right).$$

We understand the Z-transform to be taken over the discrete variable i and not the continuous variable s.

**Fig. 6:**
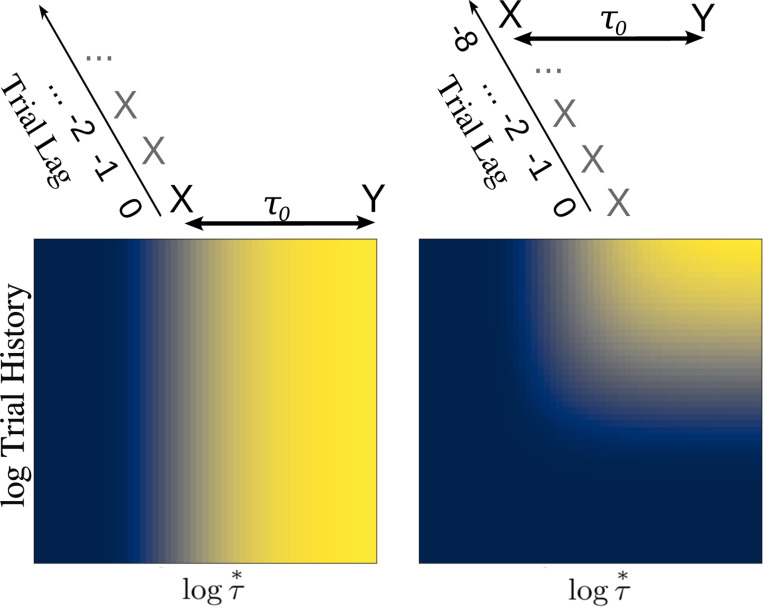
M(ρ,s) contains information about both time within a trial and trial history. Left: Consider a single pairing of x and y on the most recent trial. The heatmap shows the degree to which y is cued by x by $\frac{y^{\prime }M(\rho ,s)x}{1-\rho }$ projected onto log time. The profile as a function of logτ is identical to the profile for future time in Figure 2. If the pairing between x and y had a longer delay, the edge would be further to the right. Right: The single pairing of x and y is followed by an additional series of trials on which x was presented by itself. Now there is an edge in both trial history and time within trial. Additional trials with only x would push this edge further towards the top of the graph. Additional trials with x and y paired would be added to this plot with a time delay that reflects the timing of the pairing.

Because the Z-transform is in principle invertible, information about the entire trial history has been retained by virtue of having a continuum of forgetting rates ρ. Figure 6 illustrates the ability to extract the trial history including timing information of events that follow x from M(ρ,s)x.

This illustrates a remarkable property of Laplace-based temporal memory. Although each synaptic matrix with a specific value of $\rho_{o}$ forgets exponentially with a fixed time horizon (the time constant is given by ${\left(-\log\rho_{o}\right)}^{-1}$), the set of matrices with a continuum of ρ retains information about the *entire* trial history. Although each matrix has a specific time horizon, the set of all matrices with continuous values of ρ has a continuity of time horizons, tiling the entire trial history. In practice there must be some numerical imprecision in the biological instantiation of M(ρ,s). In principle however, a continuum of forgetting rates ρ means that the past is not forgotten. Instead the past, as a function of trial history, has been written across the continuum of ρ.

### 2.5 Estimating three point correlation functions from Z-transform

A great deal of information can be extracted from the trial histories encoded in M(ρ,s) and $\bar{M}(\rho ,s)$. $M_{z}^{y}(s)$ contains the two-point probability distribution of y and z. It would be preferable to predict the occurrence and timing of z using the three-point probability distribution.

Because M(ρ,s) contains information about the paired trial history, in principle we can extract information about the three-point correlation function. The problem of estimating the three-point correlation function between stimuli is straightforward if one has access to the trial history of both the past conditionalized on the present and the future conditionalized on the present. This information is contained in ${\bar{M}}_{x}^{y}(\rho ,s)$ and $M_{z}^{y}(\rho ,s)$ respectively.

For instance, if z only occurs on trials on which both x and y are presented, but not on trials when only one of them are presented, then we should observe a positive correlation between the trial history encoded in $M_{z}^{y}(\rho ,s=0)$ and ${\bar{M}}_{x}^{y}(\rho ,s=0)$. Similarly, one can imagine that the joint timing of the presentations of x and y predicts the timing of z, as if all three symbols are being generated by a process that can unfold at different rates.

Access to the joint statistics between symbols can in principle be leveraged to provide a much more complete prediction of the future, especially when integrated into deep networks that recode the symbols into new sets of symbols. Moreover, continuous values of ρ may allow networks built using M(ρ,s) and $\bar{M}(\rho ,s)$ to respond to non-stationary statistics.

### 2.6 Updating the future

Let us return to the problem of generating a prediction of the immediate future. We again restrict our attention to the limit as ρ goes to 1 and assume the system has experienced a very long sequence of trials with the same underlying statistics. Moreover, we assume for the present that only pairwise relationships are important, so we can neglect the temporal credit assignment problem, and construct the Laplace transform of the future that predicted solely on the basis of the present stimulus.

There are two problems that need to be resolved to write an analog of Eq. 3 for ${\text{F}}_{t+\text{Δ}t}^{+}(s)$. First, we can only use Eq. 2 to update ${\text{F}}_{t}^{+}(s)$ if ${\text{F}}_{t}^{+}(s)$ is already the Laplace transform of a predicted future; we must create a circumstance that makes that true. Second, we need to address the situation where a prediction reaches the present. Because of the discontinuity at τ=0 special considerations are necessary to allow the time of a stimulus to pass from the future to the past.

#### 2.6.1 Predicting the future with the present

Equation 8 indicates that the weights in $M_{y}^{x}(s)$ record the future occurrences of y given that x occurs in the present. $M_{y}^{x}(s)$ captures both the probability that y will follow x as well as the distribution of temporal delays at which y is expected to occur. This information is encoded as a probability times the Laplace transform of a random variable. If we only need to consider x in predicting the future, then $M_{y}^{x}(s)$ is precisely how we would like to initialize the future prediction for y in ${\text{F}}_{t}^{+}(s)$ after x is presented (Fig. 4).

We probe M(s) with the “immediate past.” When x is presented it enters ${\text{F}}_{t}^{-}(s)$ as 𝓛{δ(0)x}(s). Multiplying M(s) from the right with the immediate past, yields a prediction for the future.

(18) $$\text{M}(s)e^{-s0}\;x=\text{M}(s)x=P(y|x)\;\text{𝓛}\left\{\tau_{xy}\right\}\;(s)\;y$$

More generally, the input to the future at time t should be given by $\text{M}(s)\text{𝓛}\left\{\delta (0){\text{f}}_{t}\right\}$. For concision we write this as $\text{M}(s){\text{f}}_{t}$. Because the past stored in M(s) was a probability times the Laplace transform the distribution of a random variable, the future recovered in this way is also understandable as a probability times the Laplace transform of a random variable. If only Laplace transforms of delta functions can be represented in ${\text{F}}_{t}^{+}(s)$, then we can imagine sampling from this distribution of future times, perhaps with a preference for times more near to the future.

#### 2.6.2 Continuity of the predicted future through τ=0

The neural representation described here approximates a continuous timeline by stitching together separate Laplace neural manifolds for the past and the future. With the passage of time, information in the future moves ever closer to the present. As time passes and a prediction reaches the present, this discontinuity must be addressed. Otherwise, the firing rates will grow exponentially without bound.

We can detect predictions that have reached the present by examining ${\text{F}}_{t}^{+}(s=\infty )$, which only rises from zero when τ→0. In practice, we would use $s_{\max}$ which should be on the order of ${\left(\text{Δ}t\right)}^{-1}$. If the future that is being represented is the Laplace transform of a delta function, then we can simply take components for which ${\text{F}}_{t}^{+}\left(s_{\max}\right)>0$ to zero for all s at the next time step. More generally, if the future that is represented is not simply a delta function, the linearity of the Laplace transform allows us to subtract ${\text{F}}_{t}^{+}(s=\infty )$ from all s values without affecting the evolution at subsequent time points.

If a prediction reaches the present and is observed, then no further action is needed. If a prediction reaches the present, but is not observed, we can trigger an observation of a “not symbol”, written e.g., $\tilde{\text{x}}$ to describe the observation of a failed prediction for a stimulusx. Although we won’t pursue it here, one could allow “not symbols” to be predicted by stimuli and to predict other stimuli, allowing for the model to provide appropriate predictions for a relatively complex set of contingencies using only pairwise relationships.

#### 2.6.3 Evolving ${\text{F}}_{t+\text{Δ}t}^{+}(s)$

Integrating these two additional factors allows us to write a general expression for evolving ${\text{F}}_{t}^{+}(s)$ to ${\text{F}}_{t+\text{Δ}t}^{+}(s)$.

(19) $${\text{F}}_{t+\text{Δ}t}^{+}(s)=e^{s\text{Δ}t}{\text{F}}_{t}^{+}(s)-{\text{F}}_{t}^{+}(s=\infty )+\text{M}(s){\text{f}}_{t}.$$

If the future is expressed as a delta function, continuous attractor networks with an edge are sufficient to support this evolution (Daniels & Howard, submitted). Because the future is in general more complex than a delta function, and predictions for distant parts of the future can change as events happen in the present, additional considerations are necessary.

## 3 Neural predictions

Regions as widely separated as the cerebellum (De Zeeuw, Lisberger, & Raymond, 2021; Wagner & Luo, 2020), striatum (e.g., van der Meer & Redish, 2011), PFC (e.g., Ning, Bladon, & Hasselmo, 2022; Rainer, Rao, & Miller, 1999), OFC (e.g., Namboodiri et al., 2019; Schoenbaum, Chiba, & Gallagher, 1998; Young & Shapiro, 2011), hippocampus (Duvelle, Grieves, & van der Meer, 2023; Ferbinteanu & Shapiro, 2003) and thalamus (Komura et al., 2001) contain active representations that code for the future. One can find evidence of predictive signals extending over long periods of time that modulate firing in primary visual cortex (Gavornik & Bear, 2014; Homann, Koay, Chen, Tank, & Berry, 2022; H. Kim, Homann, Tank, & Berry, 2019; Yu et al., 2022). Prediction apparently involves a substantial proportion of the brain. Coordinating activity and plasticity over such a wide region would require careful synchronization (Hamid, Frank, & Moore, 2021; Hasselmo, Bodelón, & Wyble, 2002). The timescale of this synchronization, presumably on the order of 100 ms, fixes Δt, places a bound on the fastest timescales 1/s that can be observed, and operationalizes the duration of the “present.”

Given the widespread nature of predictive signal, we will not attempt to map specific equations onto specific brain circuits. Rather we will illustrate the observable properties implied by these equations with an eye towards facilitating future empirical work. The predictions fall into two categories. One set of predictions describes properties of ongoing firing of neurons participating in Laplace Neural Manifolds for past and future time. Another set of predictions are a direct consequence of the properties of learned weights. We also briefly discuss the model in this paper in the context of recent empirical work on the computational basis of the dopamine signal (Jeong et al., 2022).

### 3.1 Active firing neurons

This paper proposes the existence of Laplace Neural Manifolds to code for the identity and time of *future* events. This implies there should be two related manifolds, one implementing the Laplace space and one implementing the inverse space. Previous neuroscientific work has shown evidence for Laplace and inverse spaces for a timeline for the past. The properties of the proposed neural manifolds for future time can be understood by analogy to the neural manifolds for the past.

#### 3.1.1 Single-cell properties of neurons coding for the past

So-called temporal context cells observed in the entorhinal cortex (Bright et al., 2020; Tsao et al., 2018) are triggered by a particular event and then relax exponentially back to baseline firing with a variety of time constants. The firing of temporal context cells is as one would expect for a population coding $F^{-}(s)$. So-called time cells observed in the hippocampus (MacDonald et al., 2011; Pastalkova et al., 2008; Schonhaut, Aghajan, Kahana, & Fried, 2022; Shahbaba et al., 2022; Shikano, Ikegaya, & Sasaki, 2021; Taxidis et al., 2020) and many other brain regions (e.g., Akhlaghpour et al., 2016; Bakhurin et al., 2017; Jin, Fujii, & Graybiel, 2009; Mello, Soares, & Paton, 2015; Subramanian & Smith, 2024; Tiganj et al., 2018; Tiganj, Kim, Jung, & Howard, 2017) fire sequentially as events recede into the past, as one would expect from neurons participating in $\tilde{f}(\overset{*}{\tau })$ for $\overset{*}{\tau }<0$. Time cells are consistent with qualitative and quantitative predictions, including the conjecture that time constants are distributed along a logarithmic scale (Cao et al., 2022).

#### 3.1.2 Single-cell and population-level properties of neurons coding for the past and the future

**Fig. 7:**
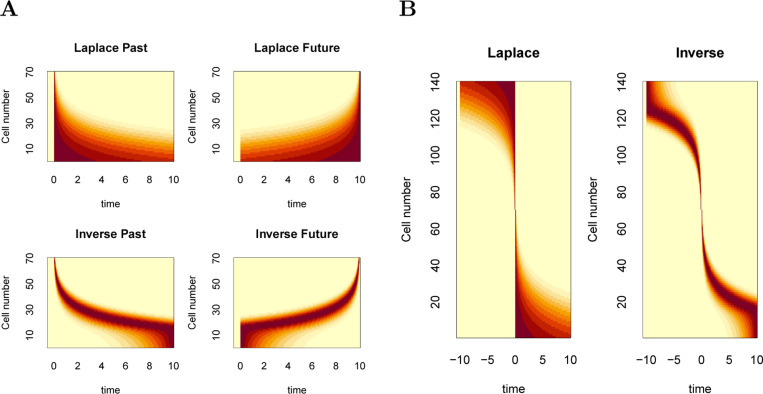
Predicted firing for Laplace and inverse spaces plotted as heatmaps. **A.** Consider an experiment in which x precedes y separated by 10 s. The top row shows firing as a function of time for cells in the Laplace space for the past (left) and the future (right). Note that the cells in $F_{t}^{-}(s)$ peak at time zero and then decay exponentially. In contrast cells in $F_{t}^{+}(s)$ peak at 10 s and ramp up exponentially. The bottom row shows firing as a function of time for cells in the Inverse space. **B.** Consider an experiment in which y is predicted to occur at time zero and then recedes into the past. Cells coding for both past and future are recorded together and sorted on the average time at which they fire. Left: For Laplace spaces, neurons in $F_{t}^{+}(s)$ are sorted to the top of the figure and neurons $F_{t}^{-}(s)$ are sorted to the bottom of the figure. Right: Inverse spaces show similar properties but give rise to a characteristic “pinwheel” shape.

In situations where the future can be predicted, $F^{+}(s)$ and $\tilde{f}\left(\overset{*}{\tau }>0\right)$ should behave as mirror images of the corresponding representations of the past. Figure 7A illustrates the firing of cells coding for a stimulus remembered in the past (left) and predicted in the future (right). Neurons participating in the Laplace space, sorted on their values of s, are shown in the top; neurons participating in the inverse space, sorted on their values of $\overset{*}{\tau }$ are shown on the bottom.

The firing of neurons constituting the Laplace space shows a characteristic shape when plotted as a function of time in this simple experiment. Neurons coding for the past are triggered shortly after presentation of the stimulus and then relax exponentially with a variety of rates. Neurons coding for the future ramp up, peaking as the predicted time of occurrence grows closer. The ramps have different characteristic time constants. Different populations are triggered by the presentation of different symbols (not shown) so that the identity of the remembered and predicted symbols as well as their timing can be decoded from populations coding ${\text{F}}^{-}(s)$ and ${\text{F}}^{+}(s)$. The largest value of 1/s in the figure is chosen to be a bit longer than the delay in the experiment, resulting in a subset of neurons that appear to fire more or less constantly throughout the delay (Enel, Wallis, & Rich, 2020).

The firing of neurons constituting the inverse space also shows a characteristic shape when plotted as a function of time in this simple experiment. Neurons tile the delay, with more cells firing early in the interval with more narrow receptive fields. The logarithmic compression of n results in a characteristic “backwards J” shape for the past and a mirror image “J” shape for the future. Again, different populations would code for different stimuli in the past and in the future (not shown) so that the identity of the remembered and predicted stimuli and their time from the present could be decoded from a population coding $\tilde{f}\left(\overset{*}{\tau }\right)$. Figure 7B shows firing that would be expected for a population that includes cells coding for the same stimulus, sayy, both in the past and the future around the time of a predicted occurrence of that symbol.

#### 3.1.3 Plausible anatomical locations for an internal future timeline

This computational hypothesis should evaluated with carefully planned analyses. However, the published literature shows evidence that is at least roughly in line with the hypothesis of neural manifolds for future time. Firing that ramps systematically upward in anticipation of important outcomes including planned movements has been observed in (at least) mPFC (Henke et al., 2021), ALM (Inagaki, Inagaki, Romani, & Svoboda, 2018), cerebellum (Garcia-Garcia et al., 2024), and thalamus (Komura et al., 2001). Komura et al. (2001) showed evidence for ramping firing in the thalamus that codes for outcomes in a Pavlovian conditioning experiment. Two recent papers show evidence that ramping neurons during motor preparation in ALM (Affan et al., 2024; Inagaki et al., 2018) and interval timing in mPFC (Cao et al., in press; Henke et al., 2021) do so with a continuous spectrum of time constants.

For instance, Cao et al. (in press), reanalyzing data originally published by Henke et al. (2021) observed the firing of neurons during the reproduction phase of an interval reproduction task. On each trial, the animal is exposed to a delay of T seconds, which must then be reproduced. Let us refer to the moment the reproduction phase begins as t=0. Now at time t<T, the beginning of the interval is τ=t seconds in the past and the planned movement is a time τ=T−t seconds in the future. Figure 9A shows that some neurons in mPFC ramped down as $e^{-st}$ with a continuum of rate constants s and other neurons ramped up as $e^{-s(T-t)}$, again with a continuum of rate constants s. There are potentially important differences between the empirical results in the paper and the theoretical model presented here—for instance many of the neurons coding for the time of the future planned movements rescaled the timecourse of their firing depending on the value of T on that trial—but the overall correspondence to the predictions described here is striking. In at least some regions, in some tasks, the ongoing firing of cortical neurons codes the time of planned future events *via* real Laplace transform of the future.

There is also circumstantial neurophysiological evidence for sequential firing leading to predicted future events as predicted by $\tilde{f}(\overset{*}{\tau })$ for $\overset{*}{\tau }>0$ coding for future events. Granule cells in cerebellum appear to fire in sequence in the time leading up to an anticipated reward (Wagner, Kim, Savall, Schnitzer, & Luo, 2017; Wagner & Luo, 2020). During performance of a task in which monkeys must perform a sequence of movements, neurons firing in sequence that decoded the time of *future* movements were observed in PFC but not in posterior parietal cortex (Watanabe, Kadohisa, Kusunoki, Buckley, & Duncan, 2023). OFC may be another good candidate region to look for “future time cells.” OFC has long been argued by many authors to code for the identity of predicted outcomes (Hikosaka & Watanabe, 2000; Mainen & Kepecs, 2009; Schoenbaum & Roesch, 2005). More recently Enel et al. (2020) showed sequential activation in OFC during a task in which it was possible to predict the value of a reward that was delayed for several seconds. Finally, it should be noted that the properties of $\tilde{f}(\overset{*}{\tau })$ over the future are a temporal analog of spatial “distance-to-goal” cells that have been observed in spatial navigation studies (Gauthier & Tank, 2018; Sarel, Finkelstein, Las, & Ulanovsky, 2017).

### 3.2 Predictions from weight matrix M(ρ,s)

#### 3.2.1 Properties of weights due to s

**Fig. 8:**
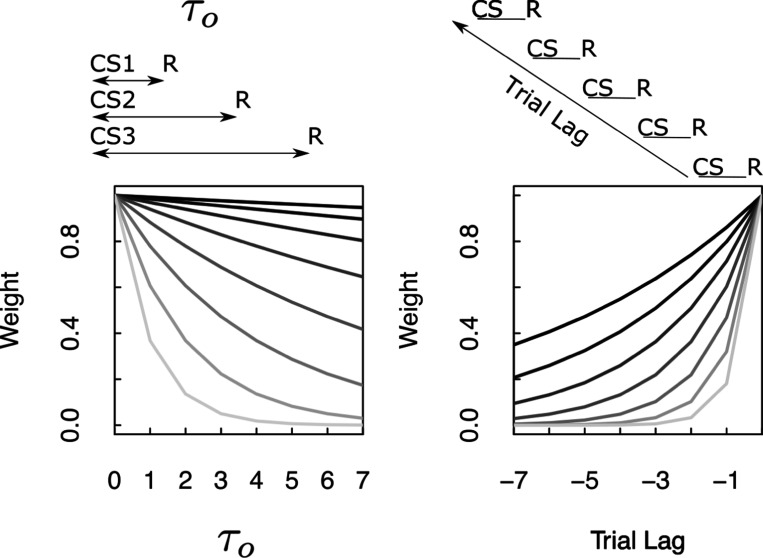
Neural predictions derived from properties of M(s). **Left.** Plot of the magnitude of the entry in ${\text{M}}_{r}(\rho =1,s)$ connecting each of the conditioned stimuli cs to the outcome r as a function of the $\tau_{o}$ corresponding to that cs. Different lines correspond to entries with different values of s. Weights corresponding to different values of s show exponential discounting as $\tau_{o}$ is manipulated, with a variety of discount rates. **Right.** Plot of the magnitude of M(ρ,s=0) associated with a single pairing of cs and r a certain number of trials in the past. Different lines show the results for different values of ρ. For clarity, these curves have been normalized such that they have the same value at trial lag zero.

Consider an experiment in which different symbols, denoted cs1, cs2, etc, precede an outcome r by a delay $\tau_{o}$. The value of $\tau_{o}$ changes across the different symbols (Figure 8A). Ignoring ρ for the moment, the strength of the connections from each cs to r depend on the value of $\tau_{o}$ for that stimulus and the value of s for each synapse: $e^{-s\tau_{o}}$. When a particular cs is presented at time t, the amount of information that flows along each synapse is $e^{-s\tau_{o}}$ and the pulse of input to ${\text{F}}_{t+\text{Δ}t}^{+}(s)-{\text{F}}_{t}^{+}(s)$ corresponding to the outcome is $e^{-s\tau_{o}}$.

**Fig. 9:**
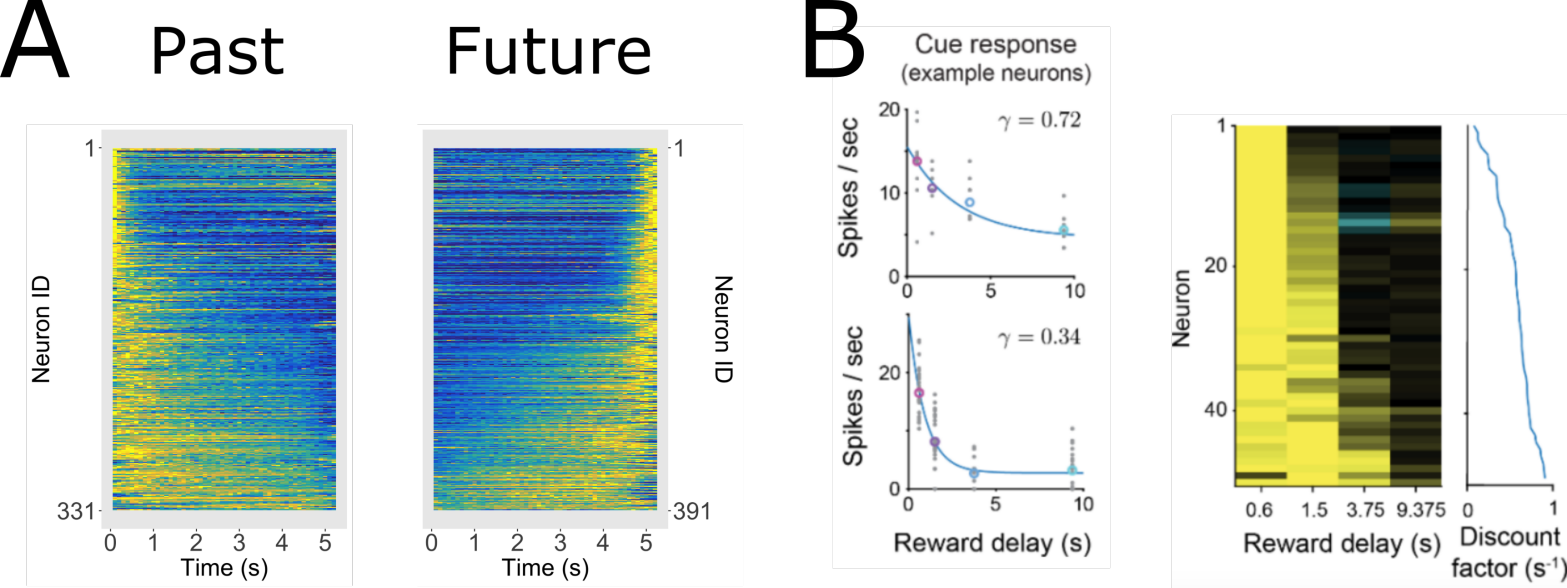
Recent observation of key neural predictions of this approach. **A.** In this interval reproduction experiment, rodents had to reproduce a delay of some duration T. Calling the start of the reproduction period t=0, at time t, the beginning of the interval is t seconds in past while the planned end of the reproduction period is T−t seconds in the future. Firing of neurons in rodent mPFC have properties resembling those predicted for $F_{t}^{-}(s)$ and $F_{t}^{+}(s)$, showing exponential decay/ramping with a continuous spectrum of time constants. Compare to Figure 7A. Adapted from Cao, et al., (2024). **B.** In a classical conditioning experiment, different stimuli predicted a rewarding outcome at different delays. Firing of dopamine neurons was recorded following each of the stimuli. The change in firing as function of the time until the reward was fitted with an exponential curve (left), indexed by the discount rate γ. Across dopamine neurons, a wide range of time constants was observed (right). The results are as one would expect if the dopamine system projects information about the time of future events to the rest of the brain *via* M(s). Adapted from Masset, et al., (2023).

Thus, considering each connection as a function of $\tau_{o}$, firing should go down exponentially as a function of $\tau_{o}$ with a rate constant that depends on the value of s. This pattern of results aligns well with experimental results observed in mid-brain dopamine neurons (Masset et al., 2023; Sousa et al., 2023). It has long been known that firing of dopamine neurons, averaged over neurons, around the time of the conditioned stimulus goes down with delay (Fiorillo, Newsome, & Schultz, 2008). Masset et al. (2023) measured the firing of dopamine neurons to different stimuli that predicted reward delivery at different delays. This study showed that there was a heterogeneity of exponential decay rates in the firing of dopamine neurons in this paradigm (Fig. 9B), much as illustrated in Fig. 8A. In the context of TDRL, this finding is consistent with a continuous spectrum of exponential discount rates (Momennejad & Howard, 2018; Tano et al., 2020). In any event, these findings (Masset et al., 2023; Sousa et al., 2023) are clear evidence that the phasic firing of midbrain dopamine neurons at the time of a predictive stimulus codes for the Laplace transform of the time until future reward.

#### 3.2.2 Properties of weights due to ρ

A continuum of forgetting rates ρ predicts a range of trial history effects. Figure 8B shows the weights in M(ρ) over past trials that result from different values of ρ. This is simply $\rho^{i}$ where i is the trial recency with values normalized such that the weight at the most recent trial is 1. The weights M(ρ) record the Z-transform of the trial history of reinforcement. Many papers show dependence on previous trial outcomes in response to a cue stimulus in learning and decision-making experiments (Akrami, Kopec, Diamond, & Brody, 2018; Bernacchia, Seo, Lee, & Wang, 2011; Hattori, Danskin, Babic, Mlynaryk, & Komiyama, 2019; Hattori & Komiyama, 2022; Morcos & Harvey, 2016; Scott et al., 2017). These studies show history-dependent effects in a wide range of brain regions and often show a continuous spectrum of decay rates within a brain region (see especially Bernacchia et al., 2011; Danskin et al., 2023). Notably, distributions of time constants for trial history effects cooccur with distributions of ongoing activity in multiple brain regions (Spitmaan, Seo, Lee, & Soltani, 2020).

### 3.3 Dopamine and learning

The connection between TDRL and neuroscience related to dopamine has been one of the great triumphs of computational neuroscience (Schultz et al., 1997). The standard account is that the firing of dopamine neurons signals reward prediction error (RPE) which drives plasticity. Despite its remarkable success at predicting the findings of many behavioral and neurophysiological experiments, the RPE account has been under increasing strain over recent years. The standard account did not predict the existence of a number of striking effects, including increasing dopamine firing during delay under uncertainty (Fiorillo, Tobler, & Schultz, 2003), dopamine ramps in spatial experiments (Howe, Tierney, Sandberg, Phillips, & Graybiel, 2013), dopamine waves (Hamid et al., 2021), and heterogeneity of dopamine responses across neurons and brain regions (Dabney et al., 2020; Masset, Malik, Kim, Bech Vilaseca, & Uchida, 2022; W. Wei, Mohebi, & Berke, 2021), although many of these phenomena can be accommodated within the RPE framework with elaboration. (Gardner, Schoenbaum, & Gershman, 2018; Gershman, 2017; H.R. Kim et al., 2020; Lee, Engelhard, Witten, & Daw, 2022) Jeong et al. (2022) reported the results of several experiments that flatly contradict the standard model. These experiments were proposed to evaluate an alternative hypothesis for dopamine firing in the brain.

Jeong et al. (2022) propose that dopamine signals whether the current stimulus is a cause of reward. The model developed there, referred to as ANCCR, assesses the contingency between a stimulus and outcomes. M(ρ,s) and $\bar{M}(s)$ contain information about the contingencies—temporal and otherwise—between a symbol and possible outcomes. Both ANCCR and the framework developed in this paper are inspired by a similar critique of Rescorla-Wagner theory and TDRL (C. Gallistel, 2021b). In order to make a complete account of the experiments in the (Jeong et al., 2022) paper, the current framework would have to be elaborated in several ways. However, the current framework does not require one to specify an intrinsic timescale of association *a priori*. Perhaps it is possible to develop a generalization of the current framework that does not rely on the simplifying assumption of discrete trials in order to yield readily interpretable measures of contingency.

## 4 Discussion

This paper takes a phenomenological approach to computational neuroscience. The strategy is to write down equations that, if the brain could somehow obey them, would be consistent with a wide range of observed cognitive and neural phenomena. The phenomenological equations make concrete predictions that can be evaluated with cognitive and neurophysiological experiments. To the extent the predictions hold, the question of how the brain manages to obey these phenomenological equations could then become a separate subject of inquiry. The phenomenological equations require a number of capabilities of neural circuits, both at the level of synapses and in terms of ongoing neural activity. We make those explicit here.

### 4.1 Circuit assumptions for synaptic weights

M(ρ,s) and $\bar{M}(\rho ,s)$ require that the brain uses continuous variables, ρ and s, to organize connections between many neurons, most likely spanning multiple brain regions. For the phenomenological equations to be viable, these continuous variables should be deeply embedded in the functional architecture of the brain. For instance, in order to invert the integral transforms, it is necessary to compute a derivative over these continuous variables. This suggests a gradient in these continuous variables should be anatomically identifiable. Conceivably anatomical gradients in gene expression and functional architecture (e.g., Guo et al., 2021; Phillips et al., 2019; Roy, Zhang, Halassa, & Feng, 2022) could generate anatomical gradients in s and/or ρ. Perhaps part of the function of traveling waves of activity such as theta oscillations (Lubenov & Siapas, 2009; Patel, Fujisawa, Berényi, Royer, & Buzsáki, 2012; Zhang & Jacobs, 2015) or dopamine waves (Hamid, Frank, & Moore, 2019) is to make anatomical gradients salient.

### 4.2 Circuit assumptions for ongoing activity

At the neural level, this framework assumes the existence of circuits that can maintain activity of a Laplace Neural Manifold over time. There is evidence that the brain has found some solution to this problem (Atanas et al., 2023; Bright et al., 2020; Cao et al., in press; Tsao et al., 2018; Zuo et al., 2023). Exponential growth of firing, as proposed by Eq. 19 seems on its face to be a computationally risky proposition (but see Daniels & Howard, submitted). However, this proposal does create testable predictions. Moreover, firing rates that increase monotonically as a function of one or another environmental variable are widely observed. For instance border cells as an animal approaches a barrier (Solstad, Boccara, Kropff, Moser, & Moser, 2008) and evidence accumulation cells (Roitman & Shadlen, 2002) both increase monotonically. If this monotonic increase in firing reflects movement of an edge along a Laplace Neural Manifold, the characteristic time scale of the increase should be heterogeneous across neurons. If the brain has access to a circuit with paired α’s, it could reuse this circuit to construct cognitive models for spatial navigation (Howard et al., 2014), evidence accumulation (Howard et al., 2018), and perhaps cognitive computation more broadly (Howard & Hasselmo, 2020). Consistent with this hypothesis, monotonic cells in spatial navigation and evidence accumulation—border cells and evidence accumulation cells—have sequential analogues (Koay, Charles, Thiberge, Brody, & Tank, 2022; Morcos & Harvey, 2016; Wilson & McNaughton, 1993) as one would expect if they reflect a Laplace space that is coupled with an inverse space.

Perhaps part of the solution to implementing these equations in the brain is to restrict the kinds of functions that can be represented over the Laplace Neural Manifold. A continuous attractor network that can maintain and evolve the Laplace transform of a single delta function per basis vector can readily be constructed (Daniels & Howard, submitted). In this case, each component of ${\text{F}}_{t}^{-}(s)$ and ${\text{F}}_{t}^{+}(s)$ would be at any moment the Laplace transform of a delta function; M(s) and $\bar{M}(s)$ would still be able to store distributions over multiple presentations. In this case when an item is presented perhaps ${\text{F}}_{t+\text{Δ}t}^{+}(s)$ could update by sampling from the distribution expressed by $\text{M}(s){\text{f}}_{t}$.

### 4.3 Generalizing beyond time

It should be possible to extend the current framework to multiple dimensions beyond time, including real space and abstract spaces (Howard et al., 2018, 2014). Properties of the Laplace domain enable data-independent operators that enable efficient computation (Howard & Hasselmo, 2020). For instance, given that a state of a Laplace neural manifold is the Laplace transform of a function, we can construct the Laplace transform of the translated function (Eq. 2, see also Shankar et al., 2016). Critically, the translation operator is independent of the function to be translated. Restricting our attention to Laplace transforms of delta functions, we can construct the sum or difference using convolution and cross correlation respectively (Howard & Hasselmo, 2020; Howard et al., 2015). The binary operators for addition and subtraction also do not need to be learned. Perhaps the control theory that governs behavior is analogous to generic spatial navigation in a continuous space.

### 4.4 Scale-covariance as a design goal

Because the s values are sampled along a logarithmic scale, all of the quantities in this paper are scale-covariant. Rescaling time, taking $\tau_{xz}\to a\tau_{xz}$, $\tau_{xy}\to a\tau_{xy}$, etc, simply takes s→s/a. Because the s values are chosen in a geometric series, rescaling time simply translates along the n axis. All the components of the model, ${\text{F}}^{-}$, ${\text{F}}^{+}$, M, and $\bar{\text{M}}$, all use the same kind of logarithmic scale for time. All of the components of the model are time scale-covariant, responding to rescaling time with a translation over cell number. Thus any measure that integrates over n (and is not subject to edge effects) is scale-invariant.

Empirically, there is not a characteristic time scale to associative learning (Balsam & Gallistel, 2009; Burke et al., 2023; C.R. Gallistel & Shahan, 2024; Gershman, 2022); any model that requires choice of a time scale for learning to proceed is thus incorrect. Logarithmic time scales are observed neurally (Cao et al., 2022; Guo et al., 2021). Logarithmic time scales can be understood as a commitment to a world with power law statistics (Piantadosi, 2016; X.-X. Wei & Stocker, 2012) or as an attempt to function in many different environments without a strong prior on the time scales it will encounter (Howard & Shankar, 2018).

Recent work has shown that the use of logarithmic time scales also enables scale-invariant CNNs for vision (Jansson & Lindeberg, 2021) and audition (Jacques, Tiganj, Sarkar, Howard, & Sederberg, 2022). For instance, (Jacques et al., 2022) trained deep CNNs to categorize spoken digits. When tested on digits presented at very different speeds than the training examples (imagine someone saying the word “seven” stretched over four seconds), the deep CNN with a logarithmic time axis generalized perfectly. Rescaling time translates the neural representation at each layer; convolution is translation equivariant; including a maxpool operation over the convolutional layer renders the entire CNN translation-invariant. Time is not only important in speech perception (e.g., Lerner, Honey, Katkov, & Hasson, 2014) but vision as well (Russ, Koyano, DayCooney, Perwez, & Leopold, 2022) suggesting that these ideas can be incorporated into a wide range of sensory systems.

### 4.5 Convolution, relational memory and cognitive graphs

There is a long-standing tension in psychology between accounts of learning based on simple associations and cognitive representations. For instance Tolman (1948) contrasted behaviorist accounts of stimulus-response associations with a “cognitive map” studying the behavior of rats in spatial mazes. This paper has already touched on this tension between association and temporal contingency—which requires metric temporal relationships between stimuli—in the study of Pavlovian learning and reward systems in the brain. (Fodor & Pylyshyn, 1988) used analogous arguments in an early critique of connectionism that echoes to the present day in contemporary debates about whether large language models “understand” language or not. Continuing interest in “neurosymbolic” artificial intelligence can be seen as an extension of this longstanding debate (Marcus, 2018).

For researchers studying episodic memory and neural representations in the hippocampus, cognitive maps rather than simple atomic associations have long been the dominant view (O’Keefe & Nadel, 1978). Cohen and Eichenbaum (1993) emphasized that cognitive maps are more general than spatial maps of the physical environment and can be used to describe other forms of relationships. In their view a *relational* representation “maintains the ‘compositionality’ of the items, that is, the encoding of items both as perceptually distinct ‘objects’ and as parts of larger scale ‘scenes’ and ‘events’ that capture the relevant relations between them.” In the view of Cohen and Eichenbaum (1993), relational memory is critical for flexible, context-dependent expression of stored knowledge, in much the same way that rats can take a novel shortcut to a reward in a pre-learned maze (Tolman, 1948). These ideas about relational memory have led to “neurosymbolic” computational models developed with specific attention to hippocampal function (Whittington et al., 2020).

The convolutions stored in the Laplace domain in M(s) and $\bar{M}(s)$ are precisely relational representations. The convolution of two functions f∗g is neither f, nor g, but is composed from them. The convolution between the function x was τ seconds in the past and the function y is in the rearward portion of the future describes an “event” including x and y in a particular relationship. If we substitute functions of physical space rather than functions of time, it would be straightforward to understand this convolution as a “scene” as proposed by Cohen and Eichenbaum (1993). Because simple Hebbian association of Laplace representations is is sufficient to perform convolution, it is straightforward to at least write down neural models for relatively complex data structures in the Laplace domain.

Not only do convolutions provide a way to implement relational representations as envisioned by Cohen and Eichenbaum (1993), they also lend themselves to flexible *expression* of memory. Convolution has an inverse operation, cross-correlation. So, if h=f∗g, then h#f≃g, where # is the cross-correlation. This property enables symbolic computation (Gayler, 2004; Schlegel, Neubert, & Protzel, 2022). For instance, consider convolutions of delta functions. If f is a delta function at $x_{f}$ and g is a delta function at $x_{g}$, then (f∗g)(x) is a delta function at $x_{f}+x_{g}$. Convolution of delta functions is thus mapped onto addition and cross-correlation—which is just convolution with reflection of one of the functions along the x axis—maps onto subtraction. With a bit of creativity to deal with positive and negative numbers, not unlike the treatment of a timeline that continues from −∞ to ∞ used here, one can build a computational system that implements the group describing the reals under addition, clearly meeting the requirements for a symbolic computer. Coming back to the hippocampus, navigation in a physical space requires vector subtraction. For instance to know how to get from physical location $x_{f}$ to physical location $x_{g}$, we must be able to compute $x_{g}-x_{f}$. One can also perform spatial navigation in abstract spaces using the same data-independent operators (C.R. Gallistel & King, 2011; Howard & Hasselmo, 2020).

Laplace Neural Manifolds are thus well-suited to not only learn, represent, and store relationships between stimuli but also to flexibly re-express relational information in a context-appropriate manner using data-independent operators. These two properties make Laplace Neural Manifolds ideal for cognitive maps of both real and abstract spaces.

## Footnotes

1 The sign convention here is distinct from prior papers that did not require τ (and $\overset{*}{\tau }$ which will be introduced shortly) to be defined on both sides of zero (Fig. 1).

2 Because of the sign conventions adopted here, $F_{t}^{-}(s)$ is the Laplace transform of $f_{t}(-\tau )$ whereas $F_{t}^{+}(s)$ is the transform of $f_{t}(\tau )$. Viewed in this light it is more precise to think of Eq. 6 as learning the Laplace transform of the cross-correlation between the present and the past.
